# Results of a long-term performance and follow-up of Endovenous Laser Ablatıon procedures performed for treating great saphenous vein incompetence

**DOI:** 10.12669/pjms.346.15683

**Published:** 2018

**Authors:** Bozkurt Gulek, Muhammet Arslan, Sinan Sozutok

**Affiliations:** 1 *Dr. Bozkurt Gulek Department of Radiology, Faculty of Medicine, University of Health Sciences, Adana, Turkey*; 2 *Dr. Muhammet Arslan Department of Radiology, Pamukkale University Faculty of Medicine, Denizli, Turkey*; 3 *Dr. Sinan Sozutok Department of Radiology, Faculty of Medicine, University of Health Sciences, Adana, Turkey*

**Keywords:** Varicose veins, Endovenous laser ablation (EVLA), Great saphenous vein, Laser efficacy, Thrombosis

## Abstract

**Objective:**

To assess the value of endovenous laser ablation (EVLA) for treating great saphenous vein (GSV) incompetence.

**Methods:**

We reviewed the overall results of EVLA procedures performed on 554 patients in our clinic between March 2011 and December 2015. Evaluations were made concerning the situations of the great saphenous vein (GSV), the energy used in the treatments, and the results obtained. We also investigated if there was a possibility to detect failure of EVLA treatment at an early stage.

**Results:**

From a total of 657 GSVs that were subjected to EVLA treatment, the procedure was found to be successful for 611 GSVs and unsuccessful for 46 GSVs (success rate: 93%). In 38 of the 46 GSVs, a thrombus formation was detected by color Doppler ultrasonography (CDUS) at the postoperative first month (82.6%).

**Conclusion:**

EVLA is a reliable and successful method utilized for the treatment of GSV incompetence. It is concluded that the detection of a thrombus in the GSV tract during the first postoperative follow-up month is an indicator for revascularization.

## INTRODUCTION

Venous incompetence of the lower extremities is a very important issue, affecting 15% males and 25% females. Insufficiencies of the great saphenous veins (GSV) majorly contribute to the venous incompetence of the lower extremities.^1^-^3^ This insufficiency may give rise to cosmetic concerns such as spider telangiectasia and varicose veins as well as to more serious problems such as limb edema, skin ulcers, and even some forms of disabilities.^4^ The main goal of therapy in GSV incompetence is to eliminate the underlying cause of venous reflux. The surgical option comprises high ligation and stripping combined with phlebectomy at the knee level.^5^ Surgery usually necessitates general anesthesia, and it may lead to certain morbidities such as hemorrhage, bleeding, pain, infection, neural damage, and scarring.^3^,^6^ EVLA is a less invasive method than surgery, and it leads to lower morbidity rates, thereby impacting the quality of life in a positive manner.^6^ EVLA, together with other endovenous thermal ablation techniques, has increasingly become a replacement for surgery. EVLA has been reported to be a successful modality, and its efficacy has been reported to be approximately 90%, in previous studies in the literature.^3^,^7^ In this retrospective study, we discuss the efficacy of EVLA in accordance with the literature. We also evaluate the impact of therapy together with the energy consumed during the procedure as well as the pre- and postoperative procedure steps utilized.

## METHODS

### Patient Selection

Patients who visited the Interventional Radiology Clinic of our hospital between March 2011 and December 2015 with complaints of limb varices, limb edema, and limb wounds were examined using color Doppler ultrasonography (CDUS). Those who were found to have GSV incompetence were recruited to undergo EVLA procedure. Prior to performing EVLA, the GSVs were examined using CDUS, and they were mapped. These maps demonstrated the lengths of the insufficient segment together with the diameter variations of the vessels.

Patients with a history of deep vein thrombosis (DVT) as well as patients suffering from deep vein insufficiencies and lower-extremity arterial diseases were excluded from the study.

### Tumescent Anesthesia and Sedation

A mixture of 20 ml of 1% lidocaine, 1 mg adrenaline, 12.5 meq bicarbonate, and 1000 cc saline solution was pumped into the perivascular spaces surrounding the affected GSVs. To perform this procedure all around the affected vessels, the tip of the application needle was maneuvered under ultrasonic guidance the needle was made to reach both the anterior and posterior sides of the vessels. Compression of the veins to achieve a thickness 3–5 mm following administration of anesthetic fluid was established as a sign of satisfactory analgesia. A majority of our patients also received sedoanalgesia, comprising intravenous injection of Midazolam and Alfentanil.

### EVLA Procedure

EVLA procedure was performed under ultrasonic guidance, immediately following sedoanalgesia and the onset of tumescence. The initial venous entrance was performed using a 21-gauge micropuncture needle set at an approximate level of 10–20 cm above the ankle. For venous entrance, we used a 0.018-inch wire and a 5-French outer catheter measuring 10 cm in length in addition to the above-mentioned 21-Gauge needle. A 0.035-inch J-tipped guidewire was passed through the needle and extended up to the saphenofemoral junction. Then, the laser sheath was introduced and passed over the guidewire, and the guidewire was extracted out. Finally, the laser wire was passed through the laser sheath. This laser wire was connected to the laser generator, which produced an 810-nm diode laser energy (MedArt A/S, Hvidovre, Denmark). The tip of the laser wire was stationarily placed at a level of approximately 1–2 cm distal to the terminal valve of the GSV. Following an optimal tumescent anesthesia, an active EVLA procedure phase was initiated and various laser energy ablations were applied to the GSV. The proximal segment of the GSV received laser energy at an intensity of 120–140 J/cm, while the mid and distal thigh portions of the vessel received 100–120 J/cm; the knee-level segment, 80-100 J/cm; and the sub-knee segment, approximately 60–80 J/cm.

Following the EVLA procedure, the patients were aided to wear a compression pantyhose and were instructed to keep wearing it for 2 days and to continue using it throughout the day for 4 weeks. The patients were also advised to walk daily for 30 minutes.

### Patient Follow-up

The patients were called for clinical follow-up and for undergoing Doppler US on the 1^st^, 3^rd^, 6^th^, 12^th^, and 24^th^ month after receiving EVLA procedure, and their health conditions were recorded.

## RESULTS

A total of 657 incompetent GSVs of 553 patients were treated by means of the EVLA procedure; 104 patients had bilateral VSM insufficiencies. The ages of the patients varied between 18 and 75 years, and their mean age was 40.5 years. Of the 553 patients, 337 patients (61%) were females, while 216 (39%) were males. Demographic data as well as GSV dimensions and ablation energies utilized during the procedures are depicted in Table-I.

**Table-I:** The demographic data of the patients, together with the pre-procedural GSV sizes, and the laser energy intensities applied during the procedures.

Number of patients	553
Number of treated GSVs	657
Age	40.5 (18–75) years
Sex	337 (61%) females; 216 (39%) males
Length of treated GSV	39 (22–65) cm
Diameter of treated GSV	10.1 (5.4–14.4) mm
Laser energy intensity	94.8 (78.2–120) J/cm

The maximum diameter of the treated GSVs was 14.4 mm, and the minimum was 5.4 mm. The lengths of the treated segments of the GSVs varied between 22 mm and 65 mm. The applied laser energy intensities varied between 268 J and 4768 J. The mean intensity of laser energy utilized was 94.8 J/cm.

At the end of 24 months, treatment to 611 GSVs was evaluated as successful and to 46 GSVs as unsuccessful. The success rate of the procedure was 93%. In 38 of these 46 GSVs, thrombus formation had been detected using Doppler US during the first postoperative month (82.6%) (Table-II).

**Table-II:** The EVLA success rate together with the frequency of encountering a thrombus during the 1^st^ month in patients whom treatment is unsuccessful, which in turn reflects the recanalization rate.

EVLA success rate	93%
Recanalization due to thrombosis	82.6%

None of our patients experienced complications such as DVT, skin burn, peripheral nerve damage, bleeding, or hematoma. One patient developed thrombophlebitis. Only 24 patients needed oral analgesics for postoperative pain. All of the patients were discharged on the day of the procedure.

Fifty-five limbs of 44 patients were subjected to additional EVLA procedures for targeting the lesser saphenous veins (LSV), the accessory veins, or the perforating veins. Moreover, sclerotherapy was performed during the 6^th^ month for treating resistant superficial varicose veins in patients in whom EVLA therapy had been unsuccessful.

## DISCUSSION

The Society for Vascular Surgery and the American Venous Forum both recommend that thermal ablation (EVLA or RFA) is a safe and effective method for the treatment of incompetent saphenous veins. US-guided foam sclerotherapy, bipolar radiofrequency (RF) ablation, cryostripping, and EVLA, are all less invasive methods compared with surgery.^8^ These modalities have a lower morbidity and recurrence rate as opposed to surgery, and their use is on the rise. The main purpose of every treatment method for incompetent saphenous veins is eradication of the underlying cause. The most frequently utilized endovascular method in this manner is EVLA. The general success rates of EVLA have been reported to be between 90% and 95%, and our results is consistent with the literature data.

During an EVLA procedure, the laser energy is absorbed by the vessel wall, and this absorption leads to tissue damage by means of photochemical and photothermolytic mechanisms. Transmural venous wall destruction causes irreversible occlusion of the vein, thereby producing a successful therapeutic result. The success of the EVLA procedure depends upon the infliction of sufficient irreversible vein wall destruction, which will in turn lead to contraction and subsequent fibrosis of the treated vein, instead of thrombosis which may give way to recanalization and thereby case treatment failure.

The amount of energy administered during the procedure may be one of the factors for unsuccessful treatment. Theivacumar et al have reported a successful GSV occlusion occurred when the energy intensity was >60 J/cm. The same authors have also reported that factors such as GSV diameter, ablated vein length, and body mass index had no effect on the outcome of therapy.^9^

Tumescent anesthesia, too, increases the success rate of EVLA, by means of compressing the wall of the vein toward the laser energy fiber. It is recommended that 10 cc of tumescent anesthesia fluid should be injected circumferentially around the vein.^10^ Tumescent anesthesia is an important procedure not only for analgesia but also for preventing neural damage.^11^ Another collateral profit of tumescent anesthesia is the reduction in the amount of blood inside the vein. This leads to better energy transfer and consequent higher energy deposition on the vessel wall. If most of this energy is absorbed by blood alone, a thrombotic occlusion takes place which may lead to recanalization even months after the treatment.

EVLA has proved to have a lower recurrence rate compared with surgical ligation and stripping. In contrast to these gold standard surgical therapies for varicose veins, endovenous treatments do not require hospitalization and general anesthesia. EVLA procedures are usually performed under tumescent anesthesia and conscious sedation. Patients treated with the EVLA procedure usually resume work during the first postoperative week.^12^

The major mechanism underlying post-procedural recanalization and reflux is thrombotic occlusion.^13^ It is believed that thrombus recanalization occurs over a period of few months, similar to that occurring during DVT. Patients who were found to have thrombosis of the treated saphenous vein during the 1-month post-operative follow-up also had partial openings in the thrombus, and this finding was accepted as the precursor for recanalization. Insufficient tumescence anesthesia, insufficient application of laser energy to the vessel wall, and improper use of the compression pantyhose may be listed among the other probable causes of thrombus formation. Because our study was a retrospective one and also because the answers obtained from the patients regarding the use of pantyhose were subjective, no objective and substantial data could be extracted concerning this issue. We believe that a larger study series designed to conduct prospective analysis is warranted to draw better conclusions on this subject.

Complications such as pain, infection, bleeding, hematoma formation, DVT, nerve damage, and skin burn may be encountered following an EVLA procedure. All of these complications, except skin burns, can also be observed after surgery. However, Rustempasic et al have reported that infection, DVT, and hemorrhage are encountered less frequently after EVLA procedures than after surgery.^14^

The structure of our study is more than competent in terms of patient population. However, we believe that large prospective studies involving other treatment modalities are needed to obtain maximum scientific data on this issue.

Thus, we would like to conclude that EVLA is a very effective and reliable method for treating GSV incompetence. The detection of a thrombus in the vein during the postoperative 1^st^ month follow-up is an indicator for recanalization.

### Authors’ Contribution

**MA, BG, SS** conceived, designed and did statistical analysis & editing of manuscript, data collection and manuscript writing.

**BG, MA** did review and final approval of manuscript.
